# Quantitative Anatomical Studies in Neurosurgery: A Systematic and Critical Review of Research Methods

**DOI:** 10.3390/life13091822

**Published:** 2023-08-28

**Authors:** Edoardo Agosti, Lucio De Maria, Pier Paolo Mattogno, Giuseppe Maria Della Pepa, Ginevra Federica D’Onofrio, Alessandro Fiorindi, Liverana Lauretti, Alessandro Olivi, Marco Maria Fontanella, Francesco Doglietto

**Affiliations:** 1 Division of Neurosurgery, Department of Surgical Specialties, Radiological Sciences, and Public Health, University of Brescia, Piazzale Spedali Civili 1, 25121 Brescia, Italy; edoardo_agosti@libero.it (E.A.); alessandro.fiorindi@asst-spedalicivili.it (A.F.); marco.fontanella@unibs.it (M.M.F.); 2 Division of Neurosurgery, Department of Clinical Neuroscience, Geneva University Hospitals (HUG), 1205 Geneva, Switzerland; 3 Department of Neurosurgery, Fondazione Policlinico Universitario A. Gemelli IRCSS, 00168 Rome, Italy; pierpaolo.mattogno@policlinicogemelli.it (P.P.M.); giuseppemaria.dellapepa@policlinicogemelli.it (G.M.D.P.); liverana.lauretti@unicatt.it (L.L.); alessandro.olivi@unicatt.it (A.O.); francesco.doglietto@policlinicogemelli.it (F.D.); 4 Department of Neurosurgery, Università Cattolica del Sacro Cuore, 20123 Rome, Italy; ginevra.federica.donofrio@gmail.com

**Keywords:** quantification, comparison, anatomical studies, neurosurgical approach, research method

## Abstract

Background: The anatomy laboratory can provide the ideal setting for the preclinical phase of neurosurgical research. Our purpose is to comprehensively and critically review the preclinical anatomical quantification methods used in cranial neurosurgery. Methods: A systematic review was conducted following the PRISMA guidelines. The PubMed, Ovid MEDLINE, and Ovid EMBASE databases were searched, yielding 1667 papers. A statistical analysis was performed using R. Results: The included studies were published from 1996 to 2023. The risk of bias assessment indicated high-quality studies. Target exposure was the most studied feature (81.7%), mainly with area quantification (64.9%). The surgical corridor was quantified in 60.9% of studies, more commonly with the quantification of the angle of view (60%). Neuronavigation-based methods benefit from quantifying the surgical pyramid features that define a cranial neurosurgical approach and allowing post-dissection data analyses. Direct measurements might diminish the error that is inherent to navigation methods and are useful to collect a small amount of data. Conclusion: Quantifying neurosurgical approaches in the anatomy laboratory provides an objective assessment of the surgical corridor and target exposure. There is currently limited comparability among quantitative neurosurgical anatomy studies; sharing common research methods will provide comparable data that might also be investigated with artificial intelligence methods.

## 1. Introduction

In recent years, evidence-based medicine has gained significant importance in surgery. In this scenario, the IDEAL (Development, Exploration, Assessment, and Long-term study) paradigm was the first promoter of evidence-based surgery [1,2,3,4]. IDEAL describes the different phases and challenges of research in surgery and includes a specific phase of preclinical research that can be performed in the anatomy laboratory. Quantitative anatomical research in neurosurgery still poses the following considerable challenges: despite the evolving innovation in surgical technologies (e.g., microscope, endoscopic-assisted techniques, robotics-assisted procedure), objective and shared methods to compare different surgical approaches are often lacking. These seem particularly important in neurosurgery, as even minor differences in surgical technique can significantly affect patient outcomes [5].

Over the last three decades, different quantitative methods have been reported in anatomical neurosurgical research. However, the heterogeneity and multitude of these methods and the different measured parameters complicate the panorama of neurosurgical anatomical quantification. This paper aims to provide a systematic and critical review of the current literature on preclinical anatomical quantification and the comparison of cranial neurosurgical approaches, analyze the proposed research methods and the studied features, and discuss their advantages and disadvantages.

## 2. Materials and Methods

### 2.1. Literature Search

The systematic review was performed per the Preferred Reporting Items for Systematic Reviews and Meta-Analysis (PRISMA) guidelines [6]. Two authors performed a systematically comprehensive literature search of the databases PubMed, Ovid MEDLINE, and Ovid EMBASE. The first literature search was performed on 10 April 2023, and the search was updated on 10 May 2023. A combination of keyword searches was performed to generate a search strategy. The search keywords, including “anatomy”, “quantification”, “neurosurgery”, “approach”, “comparison”, and “surgery”, were used in both AND and OR combinations. Studies were retrieved using the following Medical Subject Heading (MeSH) terms and Boolean operators: (“neurosurgical” OR “neurologic surgery”) AND (“approaches” OR “open” OR “microsurgery” OR “endoscopy” OR “endonasal” NOT “transorbital”) AND (“anatomy” OR “anatomical studies” OR “preclinical” OR “quantitative” NOT “qualitative”) AND (“comparison” OR “quantification” OR “methods” OR “conservative”). Other pertinent articles were identified through reference analysis of selected papers. A search filter was set to show only publications over the designated period, 1990–2023.

All studies were selected based on the following inclusion criteria: (1) English language; (2) articles that quantify and compare anatomical features of different neurosurgical approaches in the anatomy laboratory; (3) articles that quantify and compare anatomical features of different neurosurgical approaches in a virtual environment. The following exclusion criteria were employed: (1) studies that qualitatively compare surgical approaches; (2) studies reporting on neurosurgical approaches other than cranial.

The list of identified studies was imported into Endnote X9, and duplicates were removed. Two independent researchers (E.A. and L.D.M.) checked the results according to the inclusion and exclusion criteria. A third reviewer (A.F.) resolved all disagreements. Then, eligible articles were subject to full-text screening.

### 2.2. Data Extraction

For each study, we abstracted the following information: year of publication, quantified feature, quantified parameter, method, tool, and pros and cons of each technique.

### 2.3. Outcomes

Our primary outcomes were measurements related to the surgical corridor and target exposure. As for the surgical corridor, the following parameters were extrapolated from the analyzed studies: volume, surgical freedom or maneuverability, surgical window, and angle of view. Considering the target exposure, the following measurement techniques have been collected: anatomical structures visualization, linear measurements, areas, and volumes.

### 2.4. Risk of Bias Assessment

The Newcastle–Ottawa Scale (NOS) was used to assess the quality of the included studies. Quality assessment was performed by assessing the selection criteria, comparability of the study, and outcome assessment. The ideal score was 9. Higher scores indicated better quality of studies. Studies receiving 7 or more points were considered high-quality studies. Two authors performed the quality assessment independently. When discrepancies arose, papers were re-examined by the third author.

### 2.5. Statistical Analysis

Descriptive statistics were reported, including ranges and percentages. All statistical analyses were performed using the R statistical package v3.4.1 (http://www.r-project.org (accessed on 1 July 2023)).

## 3. Results

### 3.1. Literature Review

**Figure 1:**
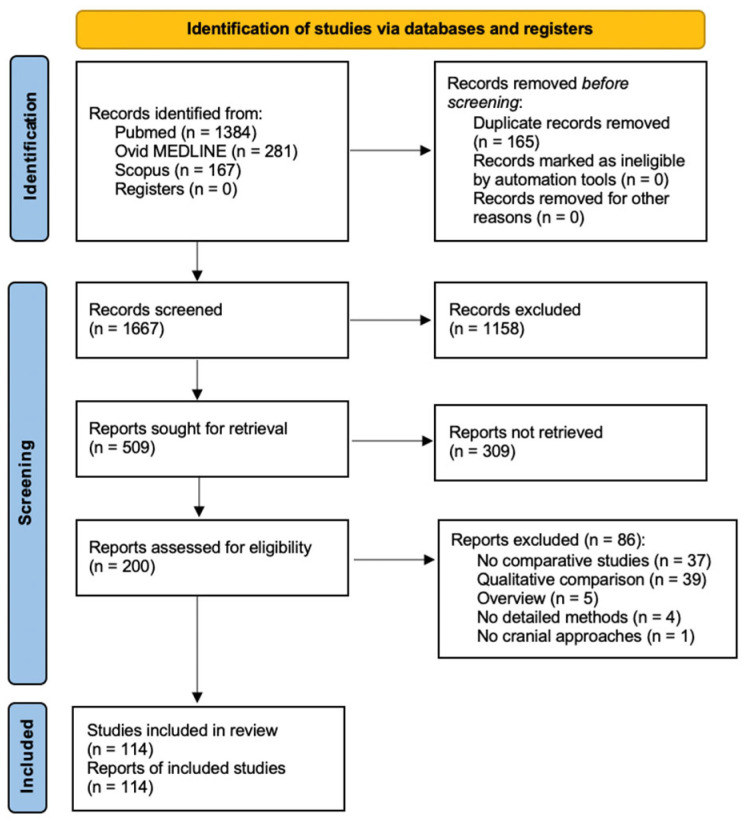
PRISMA flow diagram depicting the literature search process [6].

A total of 1667 papers were identified after duplicate removal. After title and abstract analysis, 200 articles were identified for full-text analysis. Eligibility was ascertained for 114 articles. The remaining 86 articles were excluded for the following reasons: (1) studies were not comparative (37 articles), (2) studies reporting only on qualitative comparison (39 articles), (3) overview studies (5 articles), (4) studies lacking methods details (4 articles), and (5) studies reporting on neurosurgical approaches other than cranial (1 article). All studies included in the analysis had at least one or more outcome measures available. Figure 1 shows the flow chart according to the PRISMA statement.

### 3.2. Review Data and Outcomes

A total of 114 articles were included in our systematic review. The year of publication ranged from 1996 to 2023 as follows: four articles were published before 2000 (3.5%), 17 articles were published from 2000 to 2004 (14.9%), 32 articles from 2005 to 2009 (28.1%), 29 from 2010 to 2014 (25.5%), 20 from 2015 to 2019 (17.5%), and 12 from 2020 to May 2023 (10.5%). Table 1 lists all the articles included in our review ordered per year of publication.

The surgical corridor and target were quantified on 69 articles (60.5%) and 94 articles (82.5%), respectively.

The quantified parameters of the surgical corridor were the angle of view in 42 articles (60.9%), surgical freedom or maneuverability in 20 (29%), and its volume in 16 (23.2%); the surgical window was quantified in 4 (5.8%) articles.

Target exposure was quantified by measuring the exposed area (61 articles; 64.9%) or linear distances (32 articles; 34%); semi-quantitative methods, based on visualization, were used in 14 articles (14.9%).

Table 2 and Table 3 summarize the quantified parameters, with respective methods and tools, and the advantages and disadvantages of each reported technique.

## 4. Discussion

This systematic literature review has collected and analyzed all the studies published since 1996 reporting the anatomical quantification and comparison of neurosurgical approaches.

With its constant advancements in surgical innovation and technology, neurosurgery requires objective methods to compare various surgical approaches [5,74,126,127]. Even minor differences in surgical technique can significantly impact patient outcomes in this field, and personal experience alone is no longer enough for ideal surgical decision-making. As shown by the various studies analyzed, quantifying neurosurgical approaches also aids in interpreting research results and promotes evidence-based medicine. 

**Figure 2:**
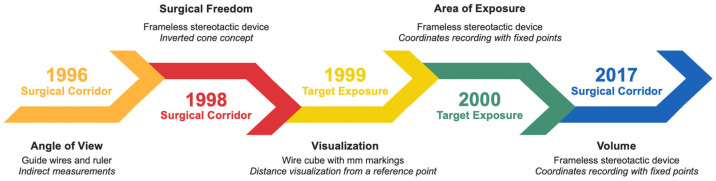
Timeline depicting the evolution of neurosurgical approaches quantification and comparison.

The systematic review revealed that, even if the first quantitative anatomical studies in neurosurgery were published in 1996, most were published after 2004 and mainly concentrated on target exposure analysis, thanks to the implementation of new technologies and dedicated software applied to preclinical research. It emerged that the quantitative measurements were initially limited to providing partial measurements of the surgical volumes, and the analysis of the surgical corridor has moved toward target exposure with the analysis of the exposure area (Figure 2).

It also emerged that the angle of view was the most frequently quantified parameter related to the surgical corridor, with 60% of the articles reporting its measurement. On the contrary, the surgical window was quantified in fewer articles, suggesting the difficulty of replicating this measurement. Regarding target exposure measurements, the exposure area was the most frequently quantified parameter, followed by linear measurements and visualization methods. These findings underscore the significance of assessing the extent of target exposure and the accuracy of surgical maneuvers during different neurosurgical approaches.

The systematic review also included a risk of bias assessment using the Newcastle–Ottawa Scale (NOS). The NOS allowed for evaluating the quality of the included studies based on selection criteria, comparability of the study, and outcome assessment. This assessment ensured that the included studies were reliable and provided robust evidence for quantifying and comparing neurosurgical approaches.

Choosing the proper research method is also paramount in quantitative anatomical studies. Direct measurements might be the best option to collect a relatively small amount of data with limited error, e.g., the length of a nerve exposed from different approaches. Neuronavigation-based methods, developed with dedicated software, allow the straightforward quantification of all the features that define the surgical pyramid, which is specific to each cranial neurosurgical approach. They can provide real-time data acquisition but also have the advantage of post-dissection data analyses, including the definition of the area of interest exposed by a specific approach.

Using standardized measurement techniques, researchers can accurately analyze and compare outcomes across different studies, enhancing the reliability and validity of their findings. This might contribute to accumulating robust evidence to guide clinical decision-making and improve patient outcomes. Furthermore, anatomical quantification facilitates the development of strategic surgical roadmaps, especially for deep-sited regions and complex targets. Additionally, quantifying neurosurgical approaches is essential for promoting new surgical strategies. For example, quantitative anatomical research has been critical in documenting the potential advantages of transnasal endoscopic transclival approaches [99,109].

While anatomical quantitative neurosurgical studies share similar research objectives, they have different research methods and are not comparable. Furthermore, despite incorporating modern technology into the research methodology, there often needs to be more adherence to scientific principles, resulting in a limited broad applicability of the findings. To address these issues, advancements in information technology and use big data analysis techniques through artificial intelligence methods are being increasingly implemented in quantitative neurosurgical anatomy research [128,129]. The final goal is to establish an evidence-based approach and achieve greater standardization and reliability in the research process.

Over the years, our research group has published several anatomical quantitative studies [96,98,99,100,106,107,109,110,113,114,116,127,130], focusing on the quantification of both the surgical volume and the exposure area. In accordance with our experience and with the aim of promoting standardization of the methods of quantification, we detail the minimum instrumentation necessary for an anatomical laboratory that wants to carry out quantitative studies. In detail:

(1) Specimens:
  - A minimum number of specimens equal to or greater than 5 so that the sample size of the data obtained allows the obtaining of statistically strong results;
  - Better alcohol-fixed specimens, as they have a greater preservation of the anatomical tissues and the respect of the relationships between the neurovascular structures, they convert more over time.
(2) Computed tomography scan:
  - 1 × 1. frame with contiguous slices, both at 1 and 3 mm;
  - Parameters: gantry of 0°, scan window diameter of at least 225 mm and pixel size of more than 0.44 × 0.44;
  - Images recorded in DICOM format.
(3) Surgical instruments and tools:
  - Microscopes;
  - Endoscope with 0° and angled optics (at least 30° and 45°);
  - Straight and curved microscopic and endoscopic instruments.
(4) Neuronavigation:
  - Radiological software (e.g., RadiAnt, Philips, OsiriX, Horos);
  - Navigation system composed by a navigation hardware and a dedicated navigation software (e.g., ApproachViewer, part of GTx-UHN—GuidedTherapeutics software developed at University Health Network—Toronto, Canada).
(5) Quantification:
  - 3D rendering software (e.g., ITK-Snap, 3D Slicer);
  - Digital surface calculator (e.g., Autodesk Meshmixer);
  - Software able to intersect surgical volume and target surface to derive the exposure area (e.g., ApproachViewer, part of GTx-UHN—GuidedTherapeutics software developed at University Health Network—Toronto, Canada).
(6) Statistical analysis:
  - Software for statistical analysis (e.g., R-Studio);
  - Ideal is the collaboration and support of a biostatistician.

## 5. Conclusions

The quantification of neurosurgical approaches can assess target exposure and different surgical corridor parameters, including volume, angle of view, surgical freedom, and surgical window. These measurements can provide valuable insights into the feasibility and effectiveness of a specific approach, helping surgeons decide the best surgical approach for a specific patient. Neuronavigation-based research methods have the advantage of being relatively straightforward in data collection while also providing the possibility of post-dissection analyses. More standardization is needed to collect data that are comparable across different studies.

## Figures and Tables

**Table 1:** List of all the articles included in our systematic review ordered per year of publication.

AUTHOR, JOURNAL	YEAR	QUANTIFIED FEATURES (Details)	SURGICAL APPROACHES
Honeybul, [7] Acta Neurochir	1996	Surgical corridor (angle of view, surgical freedom, surgical window)	Orbitozygomatic infratemporal fossa approach
Honeybul, [8] Acta Neurochir	1996	Surgical corridor (angle of view)	Extended transbasal approach
Ammirati, [9] Neurosurgery	1998	Target exposure (visualization)	Le Fort 1 approach with splitting or down-fracturing of the hard palate, extended maxillectomy, median mandibulotomy with glossotomy, mandibular swing transcervical approach
Spencer, [10] Laryngoscope	1999	Target exposure (visualization)	Transethmoidal, endonasal-trans-sphenoidal, sublabial-trans-sphenoidal approaches
Spektor, [11] J Neurosurg	2000	Target exposure (area)	Far-lateral transcondylar transtubercular approach
Horgan, [12] J Neurosurg	2000	Surgical corridor (surgical freedom, surgical window); target exposure (area, line)	Petrosal approach to the upper and middle clivus
Evans, [13] Neurosurgery	2000	Target exposure (line)	Pre- and post-anterior clinoidectomy measurements of the optic nerve, internal carotid artery, and opticocarotid triangle
Das, [14] Neurol Res	2001	Surgical corridor (volume)	Endonasal-trans-sphenoidal, sublabial-transsphenoidal, transethmoidal approaches to the sellar and parasellar region
Wanebo, [15] Neurosurgery	2001	Surgical corridor (angle of view)	Transcondylar approach to the foramen magnum
Chanda, [16] Neurosurgery	2002	Surgical corridor (angle of view); target exposure (line)	Partial labyrinthectomy petrous apicectomy approach to the petroclival region
Nanda, [17] J Neurosurg	2002	Target exposure (line)	Far-lateral approach to intradural lesions of the foramen magnum without resection of the occipital condyle
Batay, [18] Skull Base	2002	Target exposure (line)	Extended trans-sphenoidal approach by endoscope and microscope
Gonzalez, [19] Neurosurgery	2002	Surgical corridor (angle of view)	Pterional, orbitozygomatic, maxillary extension of the orbitozygomatic approach
Devlin, [20] Skull Base	2003	Target exposure (area)	Anterior distraction of the mandible without violation of the temporomandibular joint capsule, vertical ramus osteotomy of the mandible with distraction of the proximal and distal segment
Suhardja, [21] Neurosurg Focus	2003	Target exposure (area)	Retrosigmoid and transcondylar approaches to foramen magnum and lower clival meningiomas
Mortini, [22] Skull Base	2003	Target exposure (line)	Endoscopic and microscopic extended subfrontal approach to the clivus
Andaluz, [23] Neurosurgery	2003	Surgical corridor (angle of view)	Orbitopterional approach to anterior communicating artery aneurysms
Hsu, [24] J Neurosurg	2004	Target exposure (area)	Extended middle fossa approach
Youssef, [25] Neurosurgery	2004	Target exposure (line)	Frontotemporal-orbitozygomatic craniotomy with conventional trans-sylvian exposure of the upper basilar artery through the carotid-oculomotor window, added anterior clinoidectomy, ICA mobilization, and posterior clinoidectomy
Acharya, [26] J Neurol Surg B Skull Base	2004	Surgical corridor (angle of view)	Bilateral frontal craniotomy followed by fronto-orbital osteotomy
Tanriover, [27] J Neurosurg	2004	Surgical corridor (angle of view)	Transvermian and telovelar approaches to the fourth ventricle
Figueiredo, [28] Neurosurgery	2005	Surgical corridor (angle of view); target exposure (area)	Pterional, orbitopterional, and orbitozygomatic approaches to the anterior communicating artery complex before and after gyrus rectus resection
Post, [29] Neurosurgery	2005	Target exposure (line)	Trans-sylvian trans-uncal approach for upper basilar trunk aneurysm
Balasingam, [30] J Neurosurg	2005	Surgical corridor (surgical freedom); target exposure (area)	Simple transoral, transoral with a palate split, Le Fort I osteotomy, and median labioglossomandibulotomy approaches to the extracranial periclival region
Siwanuwatn, [31] J Neurosurg	2006	Surgical corridor (angle of view); target exposure (area)	Retrosigmoid, combined petrosal, transcochlear approaches to the petroclival region
Figueiredo, [32] Neurosurgery	2006	Surgical corridor (angle of view); target exposure (area)	Pterional, orbitozygomatic, mini-supraorbital approaches
Deshmukh, [33] Neurosurgery	2006	Surgical corridor (angle of view); target exposure (area)	Telovelar and transvermian approaches to the fourth ventricle
Figueiredo, [34] Neurosurgery	2006	Surgical corridor (angle of view); target exposure (area)	Transcavernous approach to interpeduncular and prepontine cisterns
Figueiredo, [35] Neurosurgery	2006	Target exposure (visualization, line, area)	Anterior petrosectomy and transcavernous approaches to retrosellar and upper clival basilar artery aneurysms
Liu, [36] Neurosurgery	2006	Surgical corridor (angle of view, surgical window); target exposure (line)	Transzygomatic extended middle fossa approach to petrous internal carotid artery
Tanriover, [37] Neurosurgery	2006	Target exposure (line)	One-piece versus two-piece orbitozygomatic approaches
Andaluz, [38] Acta Neurochir (Wien)	2006	Target exposure (line)	Pterional approaches with anterior clinoidectomy to the ophthalmic segment of the internal carotid artery
Beretta, [39] Neurosurgery	2006	Target exposure (line)	Anterior sternocleidomastoid approach, retroparotid dissection and division of the digastric muscle, section of the styloid apparatus, and mandibulotomy to expose the distal cervical internal carotid artery
Catapano, [40] J Neurosurg	2006	Target exposure (visualization)	Microscopic and endoscopic direct endonasal extended trans-sphenoidal approach
Sincoff, [41] J Neurosurg	2006	Surgical corridor (surgical freedom)	Retrosigmoid, combined petrosal, transcochlear approaches to the petroclival region
Safavi-Abbasi, [42] J Neurosurg	2007	Surgical corridor (angle of view, surgical freedom); target exposure (area)	Retrosigmoid approach
Figueiredo, [43] Neurosurgery	2007	Surgical corridor (angle of view, surgical freedom); target exposure (area)	Minipterional approach
Wu, [44] Chin Med J (Engl)	2008	Target exposure (area)	Presigmoid transpetrosal keyhole approach to petroclival region
Jittapiromsak, [45] Neurosurgery	2008	Surgical corridor (angle of view); target exposure (area)	Retrosigmoid and lateral supracerebellar infratentorial approaches along the lateral surface of the pontomesencephalic junction
Fatemi, [46] Neurosurgery	2008	Target exposure (line)	Endonasal trans-sphenoidal approach to the suprasellar and infrasellar region
Mandelli, [47] J Neurosurg	2008	Surgical corridor (angle of view); target exposure (line)	Partial labyrinthectomy petrous apicectomy approach to petroclival meningiomas
Kuriakose, [48] J Neurol Surg B Skull Base	2008	Surgical corridor (angle of view); target exposure (line)	Transtemporal and facial translocation approaches to infratemporal fossa
D’Ambrosio, [49] Neurosurgery	2008	Surgical corridor (angle of view)	Frontotemporal orbitozygomatic approach
Dzierzanowski, [50] Folia Morphol (Warsz)	2008	Surgical corridor (angle of view)	Pterional and pterional-orbitozygomatic approaches to the basilar artery bifurcation
Pillai, [51] Neurosurgery	2009	Surgical corridor (angle of view); target exposure (area)	Endoscopic and microscopic transoral approach to the craniovertebral junction
Li, [52] Zhonghua yi xue za zhi	2009	Surgical corridor (angle of view); target exposure (line, area)	Suboccipital median transcerebellomedullary fissure keyhole approach
Jittapiromsak, [53] Neurosurgery	2009	Target exposure (area)	Supracerebellar transtentorial and occipital transtentorial approaches to the medial temporal region
Chang, [54] Neurosurgery	2009	Surgical corridor (surgical freedom); target exposure (area)	Kawase’s approach and retrosigmoid approach to tumors involving both middle and posterior fossae
Filipce, [55] Neurosurgery	2009	Target exposure (area)	Endoscopic and microscopic mini-supraorbital, pterional, orbitozygomatic approaches to the anterior communicating artery complex
Doglietto, [56] Neurosurgery	2009	Target exposure (visualization)	Endonasal microscopic trans-sphenoidal, sublabial microscopic trans-sphenoidal, transmaxillary microscopic, paraseptal endoscopic trans-sphenoidal, transethmoid-pterygoid-sphenoidal endoscopic approaches to the cavernous sinus
Baird, [57] Neurosurgery	2009	Surgical corridor (angle of view)	Endoscopic endonasal, transoral, and transcervical approaches to the craniocervical junction
Alvernia, [58] Neurosurgery	2009	Surgical corridor (angle of view)	Anterior interhemispheric approach with and without complete exposure and retraction of the superior sagittal sinus
Roth, [59] Neurosurgery	2009	Target exposure (area)	Multiple endoscopic expanded endonasal and transcranial approaches to midline cranial base targets
Wu, [60] Operative Neurosurgery	2010	Surgical corridor (angle of view); target exposure (area)	Variants of the far-lateral approach with condylar fossa and transcondylar exposures
Agrawal, [61] World Neurosurg	2010	Surgical corridor (angle of view); target exposure (area)	Extraoral and transoral approaches to the craniocervical junction
Wu, [62] Neurosurgery	2010	Surgical corridor (angle of view); target exposure (visualization, area)	Trans-sylvian transchoroidal and lateral transtemporal approaches
Safavi-Abbasi, [63] Oper Neurosurg (Hagerstown)	2010	Surgical corridor (angle of view); target exposure (area)	Retrosigmoid, far-lateral approaches, and their combination
Beretta, [64] J Neurosurg	2010	Surgical corridor (angle of view); target exposure (area)	Supraorbital and transorbital minicraniotomies to the sellar and perisellar regions
Jittapiromsak, [65] Neurosurgery	2010	Surgical corridor (angle of view); target exposure (line, area)	Telovelar approach to the recesses of the fourth ventricle
Boari, [66] J Neurosurg	2010	Surgical corridor (surgical window); target exposure (line, area)	Clival and paraclival exposure in the Le Fort I transmaxillary transpterygoid approach
Zador, [67] Neurosurgery	2010	Target exposure (line, area)	Pretemporal and subtemporal approaches
Wang, [68] Acta Neurochir (Wien)	2010	Target exposure (line)	Posterior subtemporal keyhole approach combined with the transchoroidal approach to the ambient cistern
Vince, [69] J Clin Neurosci	2010	Target exposure (visualization, line)	Supracerebellar midline and paramedian approaches to the inferior colliculus
Seker, [70] World Neurosurg	2010	Target exposure (line)	Endoscopic transnasal and transoral approaches to the craniovertebral junction
Cavalcanti, [71] Neurosurgery	2010	Surgical corridor (angle of view)	Transciliary supraorbital approach
Wang, [72] J Neurosurg	2010	Surgical corridor (surgical freedom)	Posterior interhemispheric transfalx transprecuneus approach to the atrium of the lateral ventricle
Salma, [73] Neurosurgery	2011	Surgical corridor (surgical freedom, volume); target exposure (visualization)	Lateral supraorbital approach and pterional approaches
Lin, [74] World Neurosurgery	2011	Target exposure (area)	Modified temporal-occipital transtentorial transpetrosal-ridge and transpetrosal presigmoid approaches
Sabuncuoğlu, [75] Skull Base	2011	Target exposure (line, area)	Temporopolar transcavernous approach to the basilar artery apex
Kinoshita, [76] Acta Neurochir (Wien)	2011	Target exposure (line)	Transcrusal approach to the retrochiasmatic region
Russo, [77] Neurosurgery	2011	Target exposure (visualization, line)	High anterior cervical approach to the clivus and foramen magnum
Kinoshita, [78] World Neurosurg	2012	Target exposure (line)	Pterional craniotomy, with and without the removal of the supraorbital bar and the lateral orbital wall along with the sphenoid wing to access the suprachiasmatic region
Yeremeyeva, [79] J Clin Neurosci	2012	Target exposure (visualization)	Keyhole approaches to the anterior communicating artery complex
Russ, [80] World Neurosurg	2012	Target exposure (visualization, line)	Minimally invasive supracondylar transtubercular approach to the lower clivus
Tang, [81] Clin Neurol Neurosurg	2013	Surgical corridor (angle of view); target exposure (area)	Endoscopic and microscopic retrosigmoid and posterior petrosectomy approaches to the petroclival region
McLaughlin, [82] J Clin Neurosci	2013	Target exposure (line)	Extended subtemporal transtentorial approach
Guan, [83] Chin Med J (Engl)	2013	Target exposure (line)	Endoscope-assisted far lateral keyhole approach to the ventral craniocervical region
Ambekar, [84] J Neurol Surg B Skull Base	2013	Target exposure (line)	Retrosigmoid intradural suprameatal and retrosigmoid transtentorial approaches to the petroclival region
Tang, [85] Neurosurg Rev	2013	Target exposure (visualization)	Endoscopic and microscopic approaches for neurovascular decompression of the trigeminal nerve
Cheng, [86] J Neurosurg	2013	Target exposure (area)	Supraorbital keyhole, frontotemporal pterional, and supraorbital approaches to the parasellar region
Wilson, [87] World Neurosurg	2014	Surgical corridor (angle of view, surgical freedom)	Minimal-access endoscopic transmaxillary approaches to the anterolateral skull base
de Notaris, [88] Laryngoscope	2014	Surgical corridor (surgical freedom); target exposure (area)	Endoscopic suprasellar approach
Jacquesson, [89] Surg Radiol Anat	2015	Target exposure (area)	Anterior petrosectomy and expanded endoscopic endonasal approach to petroclival tumors
Jacquesson, [90] World Neurosurg	2015	Target exposure (visualization)	Anterior expanded endoscopic endonasal, retrosigmoid, anterior petrosectomy approaches to the petroclival region
Tripathi, [91] J Neurosurg	2015	Surgical corridor (angle of view, surgical freedom); target exposure (area)	Kawase versus the modified Dolenc-Kawase approaches to the middle cranial fossa
Kim, [92] Neurosurgery	2015	Target exposure (area)	Supraorbital modified orbitozygomatic approach to the opticocarotid and carotid-oculomotor windows before and after internal carotid artery mobilization and posterior communicating division
Yang, [93] Acta Neurochir (Wien)	2016	Surgical corridor (surgical freedom); target exposure (area)	Microscopic and endoscopic retrolabyrinthine and transcrusal approaches to the retrochiasmatic region
Lee, [94] Neurosurg Rev	2016	Target exposure (area)	Pterional transtentorial, orbitozygomatic, and anterior petrosal approaches to the anterosuperior pons
Jägersberg, [95] World Neurosurg	2017	Surgical corridor (volume); target exposure (area)	Pterional approach and its minimally invasive variants
Schreiber, [96] World Neurosurg	2017	Surgical corridor (volume); target exposure (area)	Modular endoscopic medial maxillectomies
Araujo, [97] J Neurosurg	2017	Surgical corridor (angle of view, volume); target exposure (area)	Transcallosal-transchoroidal and transcallosal-transforniceal-transchoroidal approaches to the third ventricle
Belotti, [98] World Neurosurg	2018	Surgical corridor (volume); target exposure (area)	Modular endoscopic endonasal trans-sphenoidal approaches to sellar region
Doglietto, [99] World J Methodol	2018	Surgical corridor (volume); target exposure (area)	Transnasal endoscopic and lateral approaches to the clivus
Muhanna, [100] J Neurol Surg B	2018	Surgical corridor (volume); target exposure (area)	Endoscopic and maxillary swing surgical approaches for nasopharyngectomy
Peraio, [101] Br J Neurosurg	2018	Surgical corridor (surgical freedom)	Supraorbital and endonasal approaches
Wu, [102] Acta Neurochir (Wien)	2018	Surgical corridor (surgical freedom); target exposure (area)	Microscopic and endoscopic far lateral approaches to the cranio-vertebral junction
Di Somma, [103] J Neurosurg	2018	Surgical corridor (surgical freedom); target exposure (area)	Endoscopic endonasal transtuberculum transplanum approach
Bozkurt, [104] World Neurosurg	2018	Surgical corridor (angle of view, surgical freedom)	Transcallosal-transchoroidal and transcallosal-subchoroidal approaches to the floor of the third ventricle
Belykh, [105] World Neurosurg	2018	Surgical corridor (angle of view, volume); target exposure (area)	Ipsilateral and contralateral interhemispheric transcallosal approaches to the lateral ventricle
Doglietto, [106] Acta Neurochir (Wien)	2019	Surgical corridor (volume); target exposure (area)	Endonasal and transoral approaches to the craniovertebral Junction
Ferrari, [107] Head Neck	2019	Surgical corridor (volume); target exposure (area)	Transnasal, sublabial, transoral, transcervical, and infratemporal approaches to the parapharyngeal space
da Silva, [108] World Neurosurg	2019	Surgical corridor (angle of view); target exposure (line, area)	Pterional, pretemporal, orbitozygomatic approaches
Agosti, [109] Acta Neurochir (Wien)	2020	Surgical corridor (volume); target exposure (area)	Endoscopic transnasal, and microsurgical supraorbital, minipterional, pterional, pterional transzygomatic, fronto-temporal-orbito-zygomatic, subtemporal, retrosigmoid, far-lateral, retrolabyrinthine, translabyrinthine, transcochlear approaches to the clivus
Saraceno, [110] World Neurosurg	2020	Surgical corridor (volume); target exposure (area)	Microsurgical supraorbital, minipterional, pterional, pterional-transzygomatic, fronto-temporal-orbito-zygomatic, subtemporal, and endoscopic transnasal, transorbital, transmaxillary approaches to the middle cranial fossa
Topczewski, [111] Acta Neurochir (Wien)	2020	Target exposure (area)	Endoscopic endonasal and transorbital approaches to the petrous apex
Martínez-Pérez, [112] J Neurosurg	2020	Surgical corridor (surgical freedom); target exposure (area)	Minipterional and supraorbital approaches
Agosti, [113] Oper Neurosurg (Hagerstown)	2021	Surgical corridor (volume); target exposure (area)	Multiple microsurgical transcranial, endoscopic endonasal, and transorbital approaches to the spheno-orbital region
Agosti, [114] Oper Neurosurg (Hagerstown)	2022	Surgical corridor (volume); target exposure (area)	Endoscopic endonasal transcribriform, transtuberculum, transplanum, and microsurgical transfrontal sinus interhemispheric, frontobasal interhemispheric, subfrontal, supraorbital, minipterional, pterional, frontotemporal orbitozygomatic approaches to the anterior cranial fossa
Houlihan, [115] Oper Neurosurg (Hagerstown)	2022	Surgical corridor (angle of view, surgical freedom)	Supraorbital and pterional approaches to paramedian vascular structures
Serioli, [116] Neurosurg Rev	2023	Surgical corridor (volume); target exposure (area)	Microsurgical transcranial approaches to the posterior surface of petrosal portion of the temporal bone
Martins Coelho, [117] World Neurosurg	2023	Surgical corridor (surgical freedom); target exposure (area)	Retrosigmoid and retrolabyrinthine posterior petrosal approaches to the petroclival region
Alexander, [118] Oper Neurosurg (Hagerstown)	2023	Target exposure (visualization)	Supracerebellar infratentorial, precuneal interhemispheric, transtentorial approaches to the cerebellomesencephalic fissure
Lin, [119] Neurosurg Rev	2023	Surgical corridor (angle of view); target exposure (area)	Endoscopic presigmoid retrolabyrinthine approach to the lateral mesencephalic sulcus
Revuelta Barbero, [120] Oper Neurosurg (Hagerstown)	2023	Surgical corridor (angle of view, surgical freedom); target exposure (area)	Endoscopic expanded retrosigmoid and far-lateral approaches to the inframeatal area

**Table 2:** Summary of different methods and tools, with corresponding pros and cons, described in the literature for quantifying surgical corridor parameters.

SURGICAL CORRIDOR	METHOD	TOOLS	PROS	CONS
**Volume**	Direct measurements	Filling the surgical cavity with dyed fat post-dissection CT, and volume quantification [73]	Provides visualization and quantification of the whole surgical volume	Requires post-CT; filling material characteristics might influence results
	Coordinates recording with fixed points	Frameless stereotactic device [95,96,98,100,106,107,109,110,113,114,116]	Provides visualization and quantification of the whole surgical volume	Requires navigation and dedicated software
**Surgical Freedom/Maneuverability**	Inverted cone concept	Virtual [49,50,121]	Multiple calculations are feasible	Requires virtual model; only for one target
		Frameless stereotactic device [7,12,30,41,42,54,71,72,122]	Multiplanar evaluation for a single target	Requires navigation and dedicated software; fixed distance (10 or 15 cm) or at craniotomy level
**Surgical Window**	Direct measurements	Guide wires and ruler [5,12]	Simple	Positioning of guide wires might not always be feasible, and it simplifies actual anatomy
		Graduated scales and calipers [66]	Simple	Positioning of guide wires might not always be feasible, and it simplifies actual anatomy
		NS [48]	Simple	Positioning of guide wires might not always be feasible, and it simplifies actual anatomy
	Indirect measurements	Coordinates recording and elaboration [5]	Possible for deep targets	Requires navigation and dedicated software
**Angle of View**	Indirect measurements	CT images analysis [15,23,64]	Possible also for deep targets; provides visualization on CT after dissection	Requires CT scan
		Guide wires and ruler (with Pythagorean theorem or tangent formula) [7,8,26,58]	Simple	Indirect (i.e., minimal error increased)
		Malleable wire and protractor [48]	Simple	Indirect (i.e., minimal error increased)
		MRI stealth visualization of trajectory intersecting plane [27]	Immediate rendering of data	Requires navigation and dedicated software and MRI
		Coordinates recording and elaboration [28,32,45,51,52,60,61,62,65,71,81]	Possible for deep targets	Requires navigation and dedicated software
	Direct measurements	Robotic microscope in the spherical mode [19,28,31,33,35,42,43,63]	Feasible for deep targets	Requires dedicated microscope; connected to a computer
		Goniometer [36,47,123]	No calculations	No feasible for deep targets
		Virtual [49,50,57]	Multiple calculations are feasible	Requires virtual model; not real

Abbreviations: CT = computed tomography; MRI = magnetic resonance imaging; NA = not available.

**Table 3:** Summary of different methods and tools, with corresponding pros and cons, described in the literature for quantifying target exposure parameters.

TARGET EXPOSURE	METHOD	TOOLS	PROS	CONS
**Visualization**	Ordinal scale	Kawashima grading [34,62,80]	Simple	Semi-quantitative
		Counting critical structures encountered [69,77]	Simple	Partial evaluation
		Modification of the Ammirati and Bernardo grading system [38,56,73,79,85]	Simple, includes grading of surgical maneuverability	Operator-dependent inter-variability
	Distance visualization from a reference point	Wire cube with mm markings [14]	Simple	It does not grade exposure but visualization if different visualizing tools are used (i.e., microscope vs. endoscope)
		Ruler [40]	Simple	It does not grade exposure but visualization if different visualizing tools are used (i.e., microscope vs. endoscope)
**Line**	Indirect measurements	Barium injection of arteries, clip positioning, X-ray, and distance measurement [25]	Virtual angiography	Requires barium injection and X-ray
		Mm2 graph paper and digital imaging software [37]	Post-dissection analysis	Requires dedicated software
	Direct measurements	Graduated scales and calipers [66]	Simple	Not always feasible for deep targets
		NS [36,39,48,69,70,77,78,84]	Simple	Not always feasible for deep targets
		Digital caliper [47,67]	Simple	Not always feasible for deep targets
		Ruler [13,17,18,25,29]	Simple	Not always feasible for deep targets
		Mm paper [22,46,76]	Simple	Not always feasible for deep targets
		Malleable surgical wire [38]	Simple	Not always feasible for deep targets
	Limits of exposure	A frameless stereotactic device with MRI [33,43,65,68,124]	Possible also for deep targets	Requires navigation and dedicated software and MRI
	Coordinates recording with fixed points	Frameless stereotactic device [12,34,52,68,75,82,83,125]	Possible for deep targets	Requires navigation and dedicated software
**Area**	Indirect measurements	CT images analysis [100,101,102,103,104,105]	Provides visualization on CT after dissection	Requires CT
	Indirect measurements	Image analysis software [20,66]	Post-dissection analysis	Requires dedicated software
	Direct measurements	Digital caliper [67]	Simple; low-cost	Only for dural and bony targets; not always feasible
		Beaded pins and ruler [21]	Simple; low-cost	Only for dural and bony targets; not always feasible
	Coordinates recording with fixed points	Frameless stereotactic device [11,12,24,28,30,31,32,33,34,35,42,43,44,45,51,52,53,54,55,60,61,62,63,64,65,75,81,95,96,98,99,100,106,107,108,109,110,111,112,113,114,115,116,117,119,120]	Possible for deep targets	Requires navigation and dedicated software
	Coordinates recording with MRI visualization	A frameless stereotactic device with MRI or CT	Provides visualization of the quantified area in 3D reconstruction	Multiplanar evaluation for a single target; Requires MRI or CT

Abbreviations: CT = computed tomography; MRI = magnetic resonance imaging.

## Data Availability

The authors confirm that the data supporting the findings of this study are available within the article.
